# Safety, pharmacokinetics, and pharmacodynamics of milvexian in healthy Japanese participants

**DOI:** 10.1038/s41598-022-08768-y

**Published:** 2022-03-25

**Authors:** Vidya Perera, Zhaoqing Wang, Susan Lubin, Takayo Ueno, Tomomi Shiozaki, Wei Chen, Xiaohui Xu, Dietmar Seiffert, Mary DeSouza, Bindu Murthy

**Affiliations:** 1 Bristol Myers Squibb, Princeton, NJ USA; 2 Bristol-Myers Squibb K.K., Tokyo, Japan

**Keywords:** Drug discovery, Cardiology

## Abstract

This randomized, double-blind, placebo-controlled, multiple ascending–dose study evaluated safety, tolerability, pharmacokinetics, and pharmacodynamics of multiple doses of milvexian, an oral small-molecule FXIa inhibitor, in healthy Japanese participants. Participants received oral milvexian daily under fasted (50 mg and 200 mg) or fed conditions (500 mg) or placebo over 14 days; 24 participants (8/cohort: 6 milvexian; 2 placebo) were planned. Due to an unblinding event, participants in one cohort (200 mg daily) were discontinued, and a second cohort enrolled; 32 participants were included in safety and pharmacodynamic analyses, and 24/32 in pharmacokinetic analyses. Milvexian up to 500 mg daily for 14 days was generally well tolerated, with no deaths, serious adverse events, or discontinuations due to adverse events. Milvexian exposure increased between 50-mg and 200-mg doses. Median T_max_ was similar with 50-mg and 200-mg doses (2.5–3.0 h) and delayed under fed conditions (500 mg, 7.0–8.0 h). Median T_1/2_ was similar across doses (8.9–11.9 h). Multiple oral milvexian administrations resulted in concentration-related prolongation of aPTT and decreased FXI clotting activity. Milvexian was generally safe and well tolerated. The pharmacokinetic and pharmacodynamic profile of milvexian demonstrates suitability for further clinical development in Japanese participants.

## Introduction

Anticoagulant and antiplatelet therapies are accepted in routine clinical care as prophylaxis against serious adverse thromboembolic events in patients with cardiovascular diseases, such as atrial fibrillation, or for patients with a history of ischemic stroke, arteriosclerosis, acute coronary syndrome, coronary artery disease, or peripheral artery disease^1–5^. However, antithrombotic therapies are also associated with an increased risk of major bleeding^6^. This underscores the need for novel therapies with an improved benefit/risk profile compared with currently available options.

The coagulation process encompasses a number of highly coordinated pathways that balance clot formation and dissolution^7^. Coagulation factors are essential for maintaining hemostasis, and their activity is mediated by thrombin, which plays an instrumental role in clot formation through the conversion of fibrinogen to fibrin and by activating platelet aggregation^7,8^. The zymogen factor XI (FXI) is converted by thrombin to active FXI (FXIa), a protease, through which a positive feedback loop enhances both thrombin formation and consolidation of coagulation^9,10^. Inhibition of key factors within the coagulation cascade is a mechanism that could be used for the prevention and treatment of thrombotic events. Spontaneous bleeding is rare in individuals with congenital FXI deficiencies, and the only clinical manifestation of FXI deficiency is a tendency for mild bleeding after a serious injury or surgery^11–13^. Preclinical models showed reductions in thrombus formation and clinical studies have demonstrated a reduced risk of adverse cardiovascular events and venous thromboembolism with FXI deficiency^14–18^. In addition, both clinical and preclinical models suggest that FXIa inhibitors can reduce thrombus formation and that normal hemostasis is not solely dependent on the FXI pathway^19–22^. Taken together, this evidence suggests that inhibition of FXIa can prevent thrombosis while preserving hemostasis^9,10,23^. Therefore, FXIa inhibitors have the potential to improve the benefit/risk profile of current anticoagulants through a safer bleeding profile in a variety of conditions where patients are predisposed to a high risk of thrombotic or bleeding events.

Milvexian is a potent orally bioavailable small molecule that binds and inhibits FXIa with high affinity and selectivity^24^. Milvexian is being developed to prevent and treat thrombotic events in multiple patient populations.

Milvexian has demonstrated antithrombotic activity in preclinical models of arterial and venous thrombosis while preserving hemostasis^25,26^. In addition, phase 1 studies showed that milvexian was generally safe and well tolerated in healthy non-Japanese participants and participants with hepatic impairment^27,28^. A phase 2 study is underway investigating the use of milvexian for the secondary prevention of major cardiovascular events in patients with acute ischemic stroke, and a phase 2 study on the use of milvexian for the prevention of venous thromboembolism events in patients undergoing total knee replacement surgery has been completed^29,30^.

Genetic variation in the metabolism of anticoagulants within ethnic groups has been well documented, underscoring the need to both demonstrate safety and to investigate the potential for dose adjustments within Japanese and other patient populations^31,32^. Thus, further exploration of the safety and pharmacokinetic (PK) properties of milvexian is necessary to support clinical development within the Japanese population. The primary objective of this study was to assess the safety and tolerability of multiple oral doses of milvexian in healthy Japanese participants. Secondary and exploratory objectives included assessment of multiple-dose PK and pharmacodynamics (PD) of milvexian.

## Methods

### Ethics

This study was conducted in accordance with Good Clinical Practice, as defined by the International Council for Harmonisation and in accordance with the ethical principles underlying European Union Directive 2001/20/EC and the United States Code of Federal Regulations, Title 21, Part 50 (21CFR50). The study was registered with ClinicalTrials.gov (NCT03224260; registered on 07/18/2017; first posted on 07/21/2017). The protocol, amendments, and participant informed consent received appropriate approval by the Independent Ethics Committee (IEC) and the Institutional Review Board (IRB) of Alpha IRB (San Clemente, CA, USA) prior to initiation of the study at the site. The IRB/IEC ensured the protection of the rights, safety, and well-being of human participants involved in the clinical investigation. Prior to the beginning of the study, all subjects provided written informed consent. The study was conducted at 1 site in the United States, and all laws and regulatory requirements were adhered to.

### Study design

This was a randomized, double-blind, placebo-controlled, multiple ascending–dose study. Participants underwent screening evaluations to determine eligibility within 21 days prior to study treatment administration. Within each panel, participants were randomized to milvexian or matched placebo according to a computer-generated randomization scheme prepared by a randomization coordinator. Randomization schedules were shipped directly to a pharmacist or other individual(s) responsible for the dispensing of blinded study treatment. Participants received once-daily study treatment from study personnel sequentially under fasted conditions at 50 mg (Cohort 1) and at 200 mg (Cohorts 2a and 2b) and under fed conditions at 500 mg (Cohort 3) on Days 1 through 14.

The dosages investigated in this study were chosen based on the results of the first-in-human study of milvexian where participants received milvexian at doses between 4 and 500 mg^28^. Dose-proportional plasma concentrations were observed between 20 and 200 mg, and milvexian was safe and well tolerated through doses up to 500 mg daily for 14 days. The 50-mg and 200-mg doses under fasted conditions and 500-mg dose under fed conditions were selected to capture the dose-proportional range as well as the upper end of the dosing range investigated in the first-in-human study.

An unblinding event resulted in early discontinuation of all participants in Cohort 2a, and the protocol was amended to include enrollment of a second group of participants who received milvexian 200 mg or placebo (Cohort 2b).

### Participants

Eligible participants included healthy (as determined by medical history, physical examination, electrocardiogram [ECG], and clinical laboratory evaluations) men and women of non-childbearing potential who were 18 to 55 years of age and had a body mass index of 18.0 to 25.0 kg/m^2^. All participants were first-generation Japanese, defined as having been born in Japan and not living outside of Japan for > 10 years, and having both parents be ethnically Japanese. Women of childbearing potential or who were breastfeeding were excluded, and participants were ineligible if there was evidence of any significant acute or chronic medical illness, current or recent (within 3 months) gastrointestinal disease, or any major surgery within 4 weeks of study treatment administration.

### Safety assessments

Safety assessments were based on medical review of adverse event (AE) reports and the results of vital sign measurements, ECGs, physical examinations, and clinical laboratory tests.

### Pharmacokinetic and pharmacodynamic assessments

The following PK parameters were derived from plasma concentration versus time data: maximum observed plasma concentration (C_max_); time of C_max_ (T_max_); area under the plasma concentration curve (AUC) from time 0 to time of last quantifiable concentration (AUC_[0-T]_); AUC in one dosing interval (AUC_[TAU]_); concentration at 24 h following dose (C_24_); terminal plasma half-life (T_1/2_); effective elimination half-life (T_1/2_ Eff AUC), which explains the degree of AUC accumulation observed on Day 14; effective elimination half-life (T_1/2_ Eff C_max_), which explains the degree of C_max_ accumulation observed on Day 14; AUC accumulation index (AI AUC), which is the ratio of AUC_(TAU)_ at steady state to AUC_(TAU)_ after first dose; and C_max_ accumulation index (AI C_max_), which is the ratio of C_max_ at steady state to C_max_ after first dose.

Activated partial thromboplastin time (aPTT) and FXI clotting activity were assessed as PD parameters.

All reported PK results were analyzed by validated liquid chromatography tandem mass spectrometry (LC–MS/MS) methods. Results were generated in analytical assays using appropriate calibration curves and quality control samples that met pre-established acceptance criteria and were conducted in compliance with applicable standard operating procedures in place at the time of analysis. LC–MS/MS assays had a lower limit of quantification of 1.00 ng/mL and an upper limit of quantification of 1000 ng/mL. PD parameters (aPTT and FXI clotting activity) were measured at LabCorp Colorado Coagulation (Englewood, CO, USA).

### Sample collection

Blood samples were obtained at screening and on Days –1, 7 and 13 (prior to dosing), and 16 for clinical laboratory evaluations. Special laboratory tests (complete blood count with differential and coagulation factors only) were obtained prior to dosing on Days 5 and 10.

Details on the timing of sample collection for PK and PD assessments can be found in Supplementary Table S1.

### Statistical analyses

Safety was assessed for all participants who received 1 dose of the study drug. The PK population was defined as all participants who received at least 1 dose of milvexian and had any available concentration–time data. The evaluable PK population was defined as all participants in the PK population with adequate PK profiles for accurate estimation of PK parameters. The PD population was defined as all participants who received any study treatment and had PD data available. Participants who received placebo in each cohort were pooled into a single placebo group.

Safety outcomes, including AEs and any AEs leading to study discontinuation or death, were summarized descriptively for all treated participants. Values for vital signs, ECGs, and clinical laboratory test results, as well as all non–serious AEs reported within 3 days of the last dose of study treatment, were recorded and summarized.

Although the number of participants was not based on statistical power considerations, administration of milvexian to 6 participants in each cohort would have provided an 80% probability of observing at least 1 occurrence of any AE that occurred with 24% incidence in the population from which the sample was drawn.

All milvexian PK data were summarized using descriptive statistics. Geometric means and coefficients of variation were presented for C_max_, C_24_, AUC_(0-T)_, AUC_(TAU)_, AI AUC, and AI C_max_. Median (minimum–maximum) values were presented for T_max_. Means and standard deviations were presented for T_1/2_, T_1/2_ Eff AUC, and T_1/2_ Eff C_max_. All measurements of PD markers (ie, aPTT and FXI clotting activity) were summarized descriptively.

All statistical calculations were performed by ICON (ICON Clinical Research, LLC, Gaithersburg, MD, USA) SAS programmers using SAS Version 9.3 (SAS Institute, Inc., Cary, NC, USA) software.

## Results

### Participants

Thirty-three participants entered the randomized portion of the study and received at least 1 dose of study treatment. Due to an unblinding event, all participants in the milvexian 200-mg group (Cohort 2a) discontinued participation early and a second group of participants who received milvexian 200 mg (Cohort 2b) was enrolled. One participant in the placebo group was discontinued after receiving 4 consecutive daily doses of placebo due to participation in multiple clinical trials; this participant was excluded from analysis and replaced. Eighteen participants were included in the PK population (excluding unblinded and early terminated participants and participants who received placebo). Thirty-two participants were analyzed in the PD population. Table 1 outlines the participant demographics and disposition.

**Table 1:** Baseline characteristics for all treated participants PK, pharmacokinetics.

Characteristic^a^	Placebo (n = 6)^b^	Milvexian 50 mg^c^ (n = 6)	Milvexian 200 mg (a)^c,d^ (n = 8)	Milvexian 200 mg^c^ (n = 6)	Milvexian 500 mg^e^ (n = 6)	Total (N = 32)
Male gender, n (%)	6 (100.0)	6 (100.0)	8 (100.0)	6 (100.0)	5 (83.3)	31 (96.9)
Age	41.0 (23–48)	35.5 (29–49)	37.0 (29–54)	29.5 (23–40)	39.0 (30–55)	36.5 (23–55)
Japanese race, n (%)	6 (100.0)	6 (100.0)	8 (100.0)	6 (100.0)	6 (100.0)	32 (100.0)
Body mass index, kg/m^2^	20.50 (19.5–23.3)	22.90 (19.6–24.2)	–	23.20 (19.3–24.3)	22.90 (21.6–23.7)	22.55 (19.3–24.3)

^a^All values presented as median (minimum–maximum) unless otherwise stated.

^b^One participant in Cohort 3 (milvexian 500 mg or placebo) assigned to placebo was discontinued early due to participation in multiple clinical trials; this participant was excluded from analysis and replaced.

^c^Milvexian 50 mg and 200 mg were administered under fasted conditions.

^d^Cohort 2a, milvexian 200 mg (a), includes unblinded and early terminated participants from Cohort 2 (milvexian and placebo participants). These participants were excluded from the evaluable PK population.

^e^Milvexian 500 mg was administered under fed conditions.

### Safety and tolerability

Administration of milvexian at doses up to 500 mg once daily for 14 days was generally safe and well tolerated. No deaths or serious AEs were reported, and no AEs led to discontinuation. All reported AEs were mild to moderate in intensity and resolved with no sequelae, and none of the AEs reported were considered by the investigator to be related to study treatment. Four participants (15.4%) who received milvexian reported at least 1 AE (Table 2). There were no clinically relevant findings or trends in clinical laboratory, ECG, vital sign, or physical examination findings.

**Table 2:** Adverse events for all treated participants.

AE, n (%)	Placebo (n = 6)^a^	Milvexian 50 mg^b^ (n = 6)	Milvexian 200 mg (a)^b,c^ (n = 8)	Milvexian 200 mg^b^ (n = 6)	Milvexian 500 mg^d^ (n = 6)	All milvexian (n = 26)
Any AE	0	0	1 (12.5)	0	3 (50.0)	4 (15.4)
Headache	0	0	0	0	2 (33.3)	2 (7.7)
Constipation	0	0	0	0	1 (16.7)	1 (3.8)
Dermatitis contact	0	0	0	0	1 (16.7)	1 (3.8)
Oropharyngeal pain	0	0	0	0	1 (16.7)	1 (3.8)
Presyncope	0	0	1 (12.5)	0	0	1 (3.8)
Rhinitis	0	0	0	0	1 (16.7)	1 (3.8)
Urticaria	0	0	0	0	1 (16.7)	1 (3.8)

AE, adverse event; PK, pharmacokinetics.

^a^One participant in Cohort 3 (milvexian 500 mg or placebo) assigned to placebo was discontinued early due to participation in multiple clinical trials; this participant was excluded from analysis and replaced.

^b^Milvexian 50 mg and 200 mg were administered under fasted conditions.

^c^Cohort 2a, milvexian 200 mg (a), includes unblinded and early terminated participants from Cohort 2 (milvexian and placebo participants). These participants were excluded from the evaluable PK population.

^d^Milvexian 500 mg was administered under fed conditions.

### Pharmacokinetics

**Figure 1:**
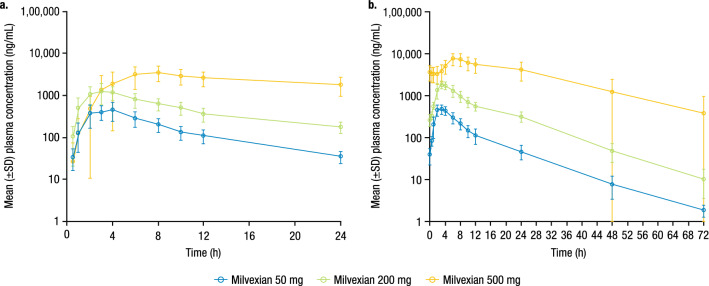
Mean (± SD) milvexian plasma concentration profile versus time by dose group on (**a**) Day 1 and (**b**) Day 14. SD, standard deviation. Milvexian 50 mg and 200 mg were administered under fasted conditions. Milvexian 500 mg was administered under fed conditions.

Milvexian exposure increased with increasing dose between the 50-mg and 200-mg doses on Days 1 through 14 (Fig. 1 and Table 3). The median T_max_ was similar for the 50-mg and 200-mg dose groups, but a delay was seen in T_max_ when milvexian 500 mg was taken with food. Specifically, median T_max_ for the 50-mg and 200-mg doses on Days 1 and 14 were 2.5 to 3.0 h compared to a median T_max_ of 7.0 to 8.0 h when milvexian 500 mg was administered with food. The mean T_1/2_ following 14 days of daily dosing was similar across doses, and the effective T_1/2_ in terms of C_max_ and AUC increased with increasing dose. Accumulation of milvexian was observed with an AI AUC of 1.14, 1.60, and 2.35, respectively, for the 50-mg, 200-mg, and 500-mg dose panels.

**Table 3:** PK parameters on Day 1 and Day 14.

Parameter^a^	Day 1			Day 14		
	Milvexian 50 mg^b^ (n = 6)	Milvexian 200 mg^b^ (n = 6)	Milvexian 500 mg^c^ (n = 6)	Milvexian 50 mg^b^ (n = 6)	Milvexian 200 mg^b^ (n = 6)	Milvexian 500 mg^c^ (n = 6)
C_max_ (ng/mL)	496 (47)	1241 (50)	4318 (31)	498 (23)	1879 (22)	7918 (29)
T_max_ (h)	3.00 (2.00–4.00)	3.00 (2.00–4.00)	8.00 (4.00–23.9)	2.50 (2.00–4.00)	3.00 (3.00–4.00)	7.00 (6.00–8.00)
AUC_(TAU)_ (ng•h/mL)	3518 (39)	11,190 (37)	50,619 (27)	4007 (29)	17,915 (22)	119,113 (37)
AUC_(0-T)_ (ng•h/mL)	–	–	–	4562 (31)	21,814 (23)	181,648 (51)
C_24_ (ng/mL)	33.9 (37)	167 (37)	1642 (52)	44.2 (42)	310 (29)	3864 (52)
T_1/2_ (h)	–	–	–	8.94 (1.67)	9.46 (1.63)	11.9 (4.60)
T_1/2_ Eff AUC (h)	–	–	–	11.9 (4.04)^d^	21.3 (11.36)^e^	31.2 (11.55)
T_1/2_ Eff C_max_ (h)	–	–	–	10.8 (3.81)^f^	20.6 (12.08)^e^	22.6 (12.89)
AI AUC	–	–	–	1.14 (27)	1.60 (42)	2.35 (27)
AI C_max_	–	–	–	1.00 (30)	1.51 (50)	1.83 (35)

PK, pharmacokinetics; C_max_, maximum observed plasma concentration; T_max_, time of maximum observed plasma concentration; AUC_(TAU)_, area under the plasma concentration curve in one dosing interval; AUC_(0-T)_, AUC from time 0 to time of last quantifiable concentration; C_24_, concentration at 24 h following dose; T_1/2_, terminal plasma half-life; T_1/2_ Eff AUC, effective elimination half-life, which explains the degree of AUC accumulation observed on Day 14; T_1/2_ Eff C_max_, effective elimination half-life, which explains the degree of C_max_ accumulation observed on Day 14; AI AUC, AUC accumulation index, which is the ratio of AUC_(TAU)_ at steady state to AUC_(TAU)_ after first dose; AI C_max_, C_max_ accumulation index, which is the ratio of C_max_ at steady state to C_max_ after first dose; %CV, coefficient of variation; SD, standard deviation.

^a^C_max_, AUC_(TAU)_, AUC_(0-T)_, C_24_, AI AUC, and AI C_max_ are presented as adjusted geometric mean (%CV); T_max_ is presented as median (minimum–maximum); T_1/2_, T_1/2_ Eff AUC, and T_1/2_ Eff C_max_ are presented as mean (SD).

^b^Milvexian 50 mg and 200 mg were administered under fasted conditions.

^c^Milvexian 500 mg was administered under fed conditions.

^d^n = 4.

^e^n = 5.

^f^n = 3.

### Pharmacodynamics

**Figure 2:**
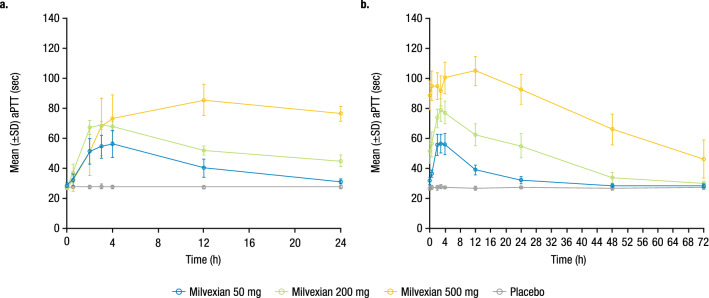
Mean (± SD) aPTT profile over time on (**a**) Day 1 and (**b**) Day 14. SD, standard deviation; aPTT, activated partial thromboplastin time. Milvexian 50 mg and 200 mg were administered under fasted conditions. Milvexian 500 mg was administered under fed conditions.

**Figure 3:**
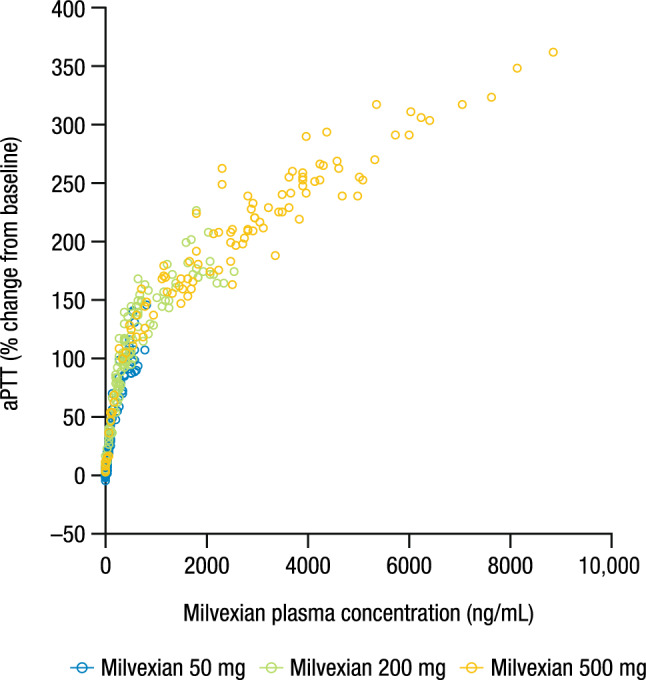
Percent change from baseline in aPTT versus milvexian plasma concentration. aPTT, activated partial thromboplastin time.

Mean baseline aPTT values were similar across groups and ranged from 27.1 to 28.6 s. Multiple doses of milvexian resulted in a concentration-related prolongation of aPTT (Fig. 2). In the fasted condition, the maximal effect was observed between 3 and 4 h postdose, which was consistent with the T_max_ of milvexian for the 50-mg and 200-mg doses. When milvexian 500 mg was administered with food, the maximal effect was delayed to 12 h postdose. The observed maximal mean percent change from baseline on Day 14 was 96.0%, 185.5%, and 286.1% for the 50-mg, 200-mg, and 500-mg doses, respectively. The relationship between aPTT and serum concentration of milvexian is shown in Fig. 3.

**Figure 4:**
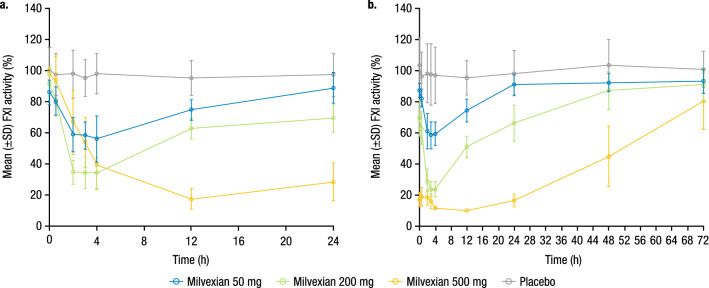
Mean (± SD) FXI clotting activity over time on (**a**) Day 1 and (**b**) Day 14. SD, standard deviation; FXI, factor XI. Milvexian 50 mg and 200 mg were administered under fasted conditions. Milvexian 500 mg was administered under fed conditions.

Mean baseline FXI clotting activity values were similar across groups and ranged from 86.0% to 100.3%. Milvexian reduced FXI clotting activity, and the magnitude of the reduction was related to the drug exposure (Fig. 4). The maximal mean decrease in FXI clotting activity from baseline on Day 14 was −31.5% and −74.1% for the 50-mg and 200-mg doses, respectively. The observed maximal effect occurred at 3 or 4 h postdose for the 50-mg and 200-mg doses, which was consistent with T_max_; however, when milvexian 500 mg was administered with food, the maximal effect was delayed to 12 h postdose.

## Discussion

This study investigated the safety, tolerability, PK, and PD of multiple ascending oral doses of milvexian in healthy Japanese participants. Milvexian is one of the first oral, small-molecule FXIa inhibitors being developed to prevent and treat thrombotic events in diverse patient populations^24^ and may offer an improvement over the benefit/risk profile of current anticoagulants. Demonstration of the safety and tolerability of milvexian in Japanese participants will allow inclusion of this population in subsequent phase 2 and 3 studies and inform whether dose adjustments will be necessary for future trials and for routine clinical care.

Milvexian was safe and well tolerated in healthy Japanese participants at doses of up to 500 mg administered with food. All observed AEs were mild in intensity, and none were considered by the investigator to be related to study treatment. There were no bleeding events reported. The safety outcomes seen here align with those from the first-in-human study of milvexian, where all treatment-emergent AEs were mild in severity and no clinically significant bleeding events were reported^28^.

Exposure to milvexian increased between the 50-mg and 200-mg doses, and there was a delay in T_max_ when milvexian 500 mg was administered with food. The PK results from the current study in Japanese participants are similar to those observed in the single- and multiple ascending–dose study in healthy non-Japanese participants. Though not directly comparable, both studies demonstrated increasing milvexian exposure as a result of increasing dose and a delay in T_max_ under fed conditions, with expected non-linearity in the PK observed at the 500-mg dose irrespective of ethnicity^28^. The rationale for allowing milvexian 500 mg to be taken with food was based on the observation from the first-in-human study that food increased milvexian exposure at doses beyond 200 mg and, thus, achieving high enough exposure to demonstrate safety and tolerability was important in this study^28^.

In Japanese participants, there was a concentration-related prolongation of aPTT and a decrease in FXI clotting activity after multiple oral administrations of milvexian, which is consistent with the mechanism of FXIa inhibition. Food intake, or potentially non-linear processes such as delayed absorption at the 500-mg dose, may have been responsible for the delayed T_max_ and extended the maximal mean effect of milvexian on aPTT and FXI clotting activity. These results are similar to those observed in a study of healthy non-Japanese participants, which showed prolongation of aPTT and a decrease in FXI clotting activity as drug exposure increased^28^. The concentration–aPTT profile observed in this study shows a direct relationship that is similar to that seen in previous studies of primarily Caucasian participants, further confirming that there are no ethnic differences in the PD relationship of milvexian in Japanese participants^28^.

Based on preclinical studies and ongoing clinical pharmacology studies, the PK and absorption, distribution, metabolism, and excretion (ADME) profiles of milvexian indicate that it is a substrate of cytochrome P450 family 3, subfamily A (also known as CYP3A) and P-glycoprotein with limited renal clearance. Although there may be inherent interindividual variability associated with these pathways, there does not appear to be distinct polymorphic variability associated with these pathways, and there do not appear to be distinct polymorphic pathways that impact the disposition of milvexian (e.g., CYP2C19 or CYP2D6). Therefore, it is unlikely that there are intrinsic pharmacogenomic factors that would play a role in determining exposure in a Japanese patient population beyond body weight.

The current study was limited by the small sample size in each panel. However, a multiple ascending–dose design allowed for the stepwise evaluation of a range of doses of milvexian in Japanese participants. In addition, this study did not include an arm of non-Japanese participants for direct comparison, leading to the potential for interstudy variability.

In conclusion, milvexian was generally safe and well tolerated in healthy Japanese participants. The PK results demonstrated an increase in exposure to milvexian between the 50-mg and 200-mg doses and that administration of milvexian 500 mg with food delayed T_max_. Milvexian resulted in concentration-related prolongation of aPTT and decrease in FXI clotting activity. Taken together, the results of this study suggest that the PK and PD profiles of milvexian in Japanese participants are similar to those observed in other populations, including other phase 1 studies in volunteers and participants with hepatic impairment^27,28^, and demonstrate suitable dosing properties for further clinical development in Japanese participants.

## Supplementary Information

Supplementary Information.

## Data Availability

The data that support the findings of this study are not publicly available due to privacy or ethical restrictions.
