# Supplementary material for: Safety, pharmacokinetics, and pharmacodynamics of milvexian in healthy Japanese participants
Source: Sci Rep. 2022 Mar 25;12:5165. doi: 10.1038/s41598-022-08768-y (PMC8956633; doi:10.1038/s41598-022-08768-y)
Supplement: Supplementary file 1 — Supplementary Information. [file 41598_2022_8768_MOESM1_ESM.pdf]

Supplementary Information

Safety, pharmacokinetics, and pharmacodynamics of milvexian in healthy Japanese participants

Vidya Perera, Zhaoqing Wang, Susan Lubin, Takayo Ueno, Tomomi Shiozaki, Wei Chen, Xiaohui (Sophia) Xu, Dietmar Seiffert, Mary DeSouza, Bindu Murthy

Supplementary Table S1. PK and PD sampling schedule for milvexian.

Study day	Time (relative to milvexian dose); hour:min	PK blood sample for plasma	PD blood sample
1	00:00	X	X
1	00:30	X	X
1	1:00	X	
1	2:00	X	X
1	3:00	X	X
1	4:00	X	X
1	6:00	X	
1	8:00	X	
1	10:00	X	
1	12:00	X	X
2	24:00	X	X
4	00:00	X	X
7	00:00	X	X
10	00:00	X	X
13	00:00	X	

14	00:00	X	X
14	00:30	X	X
14	1:00	X	
14	2:00	X	X
14	3:00	X	X
14	4:00	X	X
14	6:00	X	
14	8:00	X	
14	10:00	X	
14	12:00	X	X
15	24:00	X	X
16	48:00	X	X
17	72:00	X	X

PK, pharmacokinetics; PD, pharmacodynamics.
